# The development of a new measure of quality of life for young people with diabetes mellitus: the ADDQoL-Teen

**DOI:** 10.1186/1477-7525-2-61

**Published:** 2004-11-09

**Authors:** Carolyn V McMillan, Rachel J Honeyford, Jessica Datta, Nicola JH Madge, Clare Bradley

**Affiliations:** 1 Department of Psychology, Royal Holloway, University of London, Egham, Surrey, TW20 0EX, UK; 2 National Children's Bureau, 8 Wakley St, London, EC1V 7QE, UK

## Abstract

**Background:**

This study evaluated the psychometric properties of the ADDQoL-Teen, an innovative individualised, patient-centred questionnaire measuring perceived impact of diabetes mellitus on quality of life (QoL) of teenagers. Respondents rate all 30 life domains for frequency, and personally applicable domains for 'bother'. Two overview items measure present QoL and diabetes-dependent QoL. ADDQoL-Teen design was based on the ADDQoL (for adults with diabetes).

**Methods:**

Interviews and discussion groups were conducted with 23 teenagers aged 13–16 years, during work to design the ADDQoL-Teen. The new questionnaire was then completed by 152 young people, (mean age 16.4 ± 2.4 years), attending diabetes clinics at six UK centres.

**Results:**

Five domains detracted from the measure's reliability and factor structure, four of which were analysed separately and one deleted. The 25-domain ADDQoL-Teen had high internal consistency reliability [Cronbach's alpha = 0.91, (N = 133)] and could be summed into an overall Average Weighted Impact score. There were two subscales: a 10-item Impact-Self subscale (measuring impact of diabetes and its treatment on the individual) and a 15-item Impact-Other subscale (measuring impact on interactions with others and the external world). Both subscales had good internal consistency reliability, [Cronbach's alpha coefficients of 0.82 (N = 142) and 0.88 (N = 138) respectively]. Domains reported as most severely (and negatively) impacted by diabetes were (mean weighted impact ± SD): *lie in bed *(-3.68 ± 3.41), *interrupting activities *(-3.5 ± 3.23), *worry about the future *(-3.45 ± 3.28), *career *(-3.43 ± 3.15) and *sweets *(-3.24 ± 3.24), (maximum range -9 to +3). Analysis of the overview items showed that although 72.5% considered that their present QoL was *good *or *brilliant*, 61.8% felt that having diabetes had a negative impact on QoL, but 35.6% reported no impact and 2.6% reported a positive impact on QoL.

**Conclusions:**

The ADDQoL-Teen is a new measure of perceived impact of diabetes and its treatment on QoL of teenagers. It will help healthcare professionals and parents consider QoL issues as well as medical outcomes when caring for young people with diabetes. It may be used in clinical trials and for routine clinical monitoring in a context of continuing evaluation.

## Background

Increasing numbers of children are being diagnosed with diabetes mellitus [1] and, once diagnosed, these children and their families face major changes to their lives. However, the emphasis from health professionals is often on control of blood glucose levels and far less consideration is given to the impact of diabetes and the complex daily treatment regimen on each child's quality of life (QoL) and the child's perceptions of the disorder and its management. QoL is an outcome of diabetes management that is important in its own right and the significance of interacting biopsychosocial factors in the management of chronic disorders is recognised [2]. Thus both psychological and physiological effects of diabetes need to be measured. Measures of the impact of diabetes on the QoL of children are needed, to provide healthcare professionals with information to help protect the QoL of their patients. Such information can be used not only in research, to measure the impact of educational interventions, care provision and treatment regimens on QoL, but also in consultations where completed questionnaires can form the basis of structured discussions between the child, their parents and healthcare professionals. Professionals can be encouraged to be more patient-centred, and help children to overcome the negative impact of diabetes and its treatment on their QoL.

Adults may not be able to assess a child's point of view adequately, so children themselves should rate their own QoL wherever possible [3-5], and a more child-centred approach helps clinicians to treat patients successfully [4] and has produced data which are both valid and informative [6,7]. The questions asked in diabetes-specific adult measures such as the ADDQoL [8,9] are usually too abstract for younger children and/or inappropriate. Existing paediatric diabetes-specific QoL measures do not allow each child to say which aspects of diabetes matter to them personally: they are not sufficiently child-centred or individualised. For example the Diabetes Quality of Life Measure for adults [10] was simplified and modified to be suitable for adolescents [11], but children were not involved in the generation of items. The PedsQL [12], whilst completed by the children themselves, does not use an individualised approach, i.e. it is not possible for the individual to indicate the relevance or importance of a specific aspect of life to his or her QoL.

This paper describes the design and subsequent psychometric validation of a new teenager-centred, individualised measure of the impact of diabetes on the QoL of teenagers, the ADDQoL-Teen. The ADDQoL-Teen follows the philosophy underpinning the individualised ADDQoL measure for adults, but ideas in the teenager version are more specific and concrete than the broader, more abstract concepts of the adult version.

## Methods

### I. Design of the ADDQoL-Teen questionnaire

Four hospitals in the Greater London area participated in the research to design the questionnaire, following Ethical Committee approval. To help identify QoL issues for inclusion in the ADDQoL-Teen measure, clinic sessions were observed, health professionals consulted, and a literature review undertaken. Semi-structured interviews using open-ended questions were then conducted with 10 teenagers with diabetes, and discussions took place with 13 teenagers in small groups of 2–4 teenagers each. The views of 23 young people, aged 13–16 years, were obtained in all. The groups were single-sex as teenagers might be inhibited from talking freely about sensitive issues with members of the opposite sex present.

This qualitative research identified important QoL issues that formed the content of 30 items in the new ADDQoL-Teen questionnaire. The items were designed to reflect the teenagers' own perceptions of life with diabetes and measure their individual feelings about the importance of the issues in their everyday lives, rather than being based on researchers' or professionals' opinions. The questionnaire items, response choices and format were based on comments from the teenagers to ensure that the items were child-centred and had face validity for the teenagers themselves. The design of a questionnaire for teenagers was part of a wider study to design child-centred questionnaires for children with diabetes in three age ranges including 5–8 years (ADDQoL-Junior) and 9–12 years (ADDQoL-Junior Plus) [13].

#### Description of the ADDQoL-Teen questionnaire

**Figure 1:**
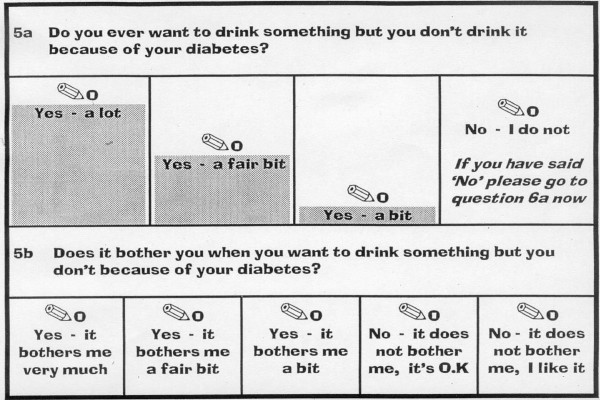
Example of an ADDQoL-Teen domain item.

In order to produce an individualised questionnaire, the ADDQoL measure of the impact of diabetes on QoL of adults [8] measures the impact of diabetes on each aspect of life and the importance of that aspect for the QoL of the individual. Design of the ADDQoL was, in turn, influenced by the generic individualised interview measure, the SEIQoL (the Schedule for the Evaluation of Individual Quality of Life) [14]. In the ADDQoL, adults' impact ratings for each applicable aspect of life (domain) are multiplied by importance ratings to provide a weighted impact score for each domain. In the new ADDQoL-Teen, however, teenagers are asked about the *frequency *('a' stem) with which diabetes impacts on each aspect of life, and then how much that particular domain *bothers *them ('b' stem). The majority of stem 'a'/frequency items are in the format: *Do you ever *..... *because of your diabetes? *and stem 'b'/bother items in the format: *Does it bother you when *..... *because of your diabetes? *(See example in Fig. 1). Multiplying frequency and bother ratings for each domain gives a domain weighted impact score. Each item provides an assessment in stem 'a' of whether the aspect of life described is relevant to the teenager, and thus contains a 'no' response option as well as multiple 'yes' response options. Stem 'a'/frequency scoring is 3, 2, 1, 0 (from *Yes *– *a lot*..... *No *– *I do not*). Stem 'b'/bother has response options scoring from -3, -2, -1, 0 (*Yes *– *it bothers me very much*..... *No *– *it does not bother me*, *it's OK*) and a positive response option (*No *– *it does not bother me*, *I like it*) scoring 1.

The ADDQoL-Teen questionnaire has 30 items dealing with specific life domains, in which the wording of item stems and response choices is appropriate to teenagers. Table 1 contains a full description of the wording of each 'a'/frequency stem as well as the item abbreviations that will be used throughout this article. The majority of items have a negative sense but there are three items (7: *extra things*, 13: *out of fix*, and 30: *holidays*) that have a positive sense. The 'b'/bother stem is scored differently for these three positive items. For example, the responses to item 7b (*How do you feel about having extra things because of your diabetes?*) are scored 3, 2, 1, 0, -1 (from *I like having them very much*......*I don't like having them*).

**Table 1:** ADDQoL-Teen item wording and abbreviations

**No:**	**Abbreviation**	**Overview item**
A	*present QoL*	In general, I feel my quality of life is.....
B	*diabetes-dependent QoL*	Does diabetes usually make your quality of life worse or better?
		**Full item as in the 'a'/frequency stem**
1	*others fuss*	Do you ever feel people fuss or worry about you because of your diabetes?
2	*sweets*	Do you ever feel you want to eat sweets but don't because of your diabetes?
3	*drink*	Do you ever want to drink something but you don't drink it because of your diabetes?
4	*eat*	Do you ever want to eat something but you don't eat it because of your diabetes?
5	*insulin*	Do you take insulin?
6	*bleed*	Do you ever bleed or have any bruises or lumpy bits where you take your insulin?
7	*extra things	Do you ever have extra things, like snacks, money, treats or days out because of your diabetes?
8	*interrupt do*	Do you ever find diabetes interrupts what you are doing, like watching TV, working at home or school, playing computer games or any other activities?
9	*finger tests*	Do you have finger prick blood tests?
10	*control*	Do you ever feel you want to take more control of diabetes on your own, with less help from other people?
11	*moody*	Do changes in your blood sugars ever make you feel moody?
12	*unwell*	Do you ever feel unwell because of your diabetes, like having a headache or pain, or feeling tired, sick or dizzy?
13	*out of fix	Do you ever find that having diabetes gets you out of a fix, or gets you out of doing something you don't want to do?
14	*sleep away*	Do you ever get asked to sleep away from home or at a friend's house, but you don't because of your diabetes?
15	*wake nights*	Do you ever wake up in the night feeling hypo with low blood sugar?
16	*lie in bed*	Do you ever want to have a lie in bed, but you don't because of your diabetes?
17	*miss events*	Do you ever miss a party, a school trip, going out or any other event because of your diabetes?
18	*low BG*	Do you ever feel your blood sugar is too low?
19	*high BG*	Do you ever feel your blood sugar is too high?
20	*worry future*	Do you ever worry about the future, like getting married, having children or your future health because of your diabetes?
21	*career*	Do you ever feel that having diabetes will make a difference to your future job or career?
22	*different*	Do you ever feel 'different' because of your diabetes?
23	*not allowed*	Are you ever told that things are 'not allowed' because of your diabetes?
24	*family life*	Do you ever feel that diabetes makes a difference to life with your family or the people you live with?
25	*responsibility*	Do you ever find you are expected to take more responsibility than you would like because of your diabetes?
26	*play sport*	Do you ever find that having diabetes makes any difference to playing sport?
27	*go toilet*	Do you ever find that you need to go to the toilet too often because of your diabetes?
28	*social life*	Do you ever find you need to fit diabetes into your social life, like carrying equipment, planning when to eat, or where to take insulin when away from home?
29	*clinic visits*	Do you go to a diabetes clinic?
30	*holidays	Have you ever been to B.D.A holidays or weekends away, or made new friends because of your diabetes?

*positive item.

B.D.A: British Diabetic Association (now known as Diabetes UK).

Finally there are two overview/global items: QA: *present QoL *and QB: *diabetes-dependent QoL*. QA (*In general, I feel my quality of life is *--- *brilliant*---*good*---*OK*---*not OK*---*bad*) is scored 3, 2, 1, -1, -2 respectively. QB (*Does diabetes usually make your quality of life worse or better? *---*a lot worse*---*a fair bit worse*---*a bit worse*---*neither worse nor better*---*better*) is scored -3, -2, -1, 0, 1. There is a free comments section at the end of the questionnaire where respondents are asked if there is anything else they would like to say about their life with diabetes.

#### Weighting the items and summation to an Average Weighted Impact score

Negative items: The 'a'/frequency ratings in categories scoring 1, 2, and 3 are multiplied by the corresponding 'b'/bother ratings to give a weighted score from -9 to +3 (maximum negative to maximum positive impact of diabetes on a domain). Zero scores on the 'a'/frequency rating are ignored as these items are not applicable to the individual and no 'b'/bother rating is made. The overall ADDQoL-Teen Average Weighted Impact score (ADDQoL-Teen AWI) can be calculated by summing weighted impact scores for all applicable domains before dividing by the number of domains applicable to the individual teenager. ADDQoL Teen AWI varies from -9 to +3, the maximum negative to maximum positive weighted impact of diabetes on overall QoL.

Positive items: items 7, 13, and 30 have weighted scores from -3 to +9 (maximum negative to maximum positive impact of diabetes on that domain). The weighting procedure for positive items is similar to that for negative items.

Overview items: QA and QB are not included in the calculation of AWI, but analysed individually.

### II. Study to determine the psychometric properties of the ADDQoL-Teen

#### Patient recruitment

In order to determine the psychometric properties of the 30-item ADDQoL-Teen, at least 150 completed questionnaires were needed, as factor analyses ideally require five or more respondents per item [15]. Young people with Type 1 diabetes mellitus (N = 78) were recruited to an interview study conducted by the National Children's Bureau [16], and also completed the questionnaire. Another 74 young people were recruited to complete the questionnaire only. Six UK centres were involved, (Centres A to F), of broad geographical spread, and serving diverse communities. Recruitment was undertaken by diabetes specialist nurses.

The criteria for inclusion were: the patient was expected to move from paediatric to adult care in the following year or the patient had moved from paediatric to adult care in the previous year. Moving from paediatric to adult care was defined as moving out of the care of the paediatric team. Depending on the size of the caseload in each research area, the nurse either included all patients or a random sample that fitted the criteria in the sampling frame. Ethical Committee approval was obtained for the study to be conducted at all the centres.

#### Statistical analyses

##### The 'No – I do not' response option and loss of data

None of the data from any respondent who selected a *No *– *I do not *response option (i.e. not applicable, hereafter referred to as "N/A") would normally be included in factor and reliability analyses, as the SPSS statistical package treats N/A responses as missing. Furthermore, if the SPSS default of listwise deletion of missing data is used, all cases that have *any *missing values across all 30 items are lost to analysis. Results of reliability and factor analyses are therefore reported below with SPSS set to pairwise deletion of missing data, and N/A responses to read as zero, to avoid considerable loss of data. This procedure has been fully described for the original development of the ADDQoL for adults [8].

##### Homogeneity of the patient sample

There was a risk of systematic differences in responses from the six UK centres creating artefactual correlations within a data set combined to provide sufficient numbers for the psychometric analyses. To check that the final sample was sufficiently homogenous ADDQoL-Teen weighted item scores were converted to standardised z scores for each subgroup, and then recombined. All questionnaire items were forced onto one factor in a Principal Components Analysis of (1) raw weighted scores and (2) recombined z scores, and results compared (a procedure used previously in the original development of the ADDQoL [8]).

##### Normality issues

Normality of distributions was investigated through histograms, box plots and standardised z(skew) values, whereby acceptable z(skew) values between ±2.58 indicate normality [17]. The ADDQoL-Teen is not a questionnaire where a normal spread of scores and normal distributions would be expected. Respondents were expected to report predominantly negative effects of diabetes with few indicating that diabetes had some positive effects on their lives. The ADDQoL-Teen identifies individuals with extreme responses – the ones most affected by their health condition. Although normality of data is desirable for factor analyses, finding transformations for skewed variables, where N/A was set to zero, that did not adversely affect normal distributions of other items in the questionnaire, proved difficult. The assumption was made that if reliability were high, the factor analysis robust, and the number of respondents sufficiently high, then a degree of non-normality was tolerable. Factor analyses were conducted on data with reflect and inverse transformations, but reliability of non-transformed variables is reported.

##### Internal consistency reliability

Cronbach's alpha coefficient [18] was determined in reliability analyses. Nunnally [19] regarded an alpha of 0.9 as the minimum acceptable for making decisions about individuals, but 0.8 adequate for comparing groups. Others consider that an acceptable minimum alpha can be 0.7 – 0.8, or even lower for short subscales [20]. In the present analyses a minimum alpha of 0.9 was regarded as ideal, but alphas above 0.8 were considered very acceptable. Acceptable corrected item-total correlations were those ≥0.2 [21].

##### Factor structure

Factor structure was explored with Principal Components Analysis, using Varimax rotation. A forced one-factor solution was obtained to confirm the validity of calculating the ADDQoL-Teen AWI score, and unforced analysis to investigate the existence of any subscales. Salient loadings were taken as ≥0.4, higher than the recommended minimum 0.3 [22], erring on the side of caution in an effort to reduce the risk of spurious loadings that owed their origin to non-normality of item distributions, and also to avoid multiple loadings.

##### Bonferroni correction

In exploratory investigations of correlations and subgroup differences in responses, the Bonferroni correction for familywise error was adopted (i.e. alpha was set initially to 0.05/*n *where *n *was the number of variables within a "family") and then the Holm's sequential Bonferroni procedure for multiple tests was applied [23].

##### Assessing tolerance of missing data

To assess the effects of respondent missing data on the measure's reliability, reliability analyses were run sequentially deleting the strongest item each time, (i.e. deleting the item having the lowest "alpha if item deleted" and therefore contributing most to the internal consistency reliability of the scale, as described elsewhere [24].

Analysis was conducted using SPSS for Windows (Release 9).

## Results

### Patient sample

Diabetes services have introduced age-appropriate clinics for teenagers with diabetes to help their transition from paediatric to adult care [16]. Some services offer adolescent clinics for 14–16 year olds, followed by transition clinics run jointly by paediatric and adult services. Other services run young person's clinics for young people up to 25 or 30 years. The ADDQoL-Teen was originally designed for age range 13–16 years, with the intention that it could be completed by those aged 17–18 years, many of whom would still be attending school. However, 28 of the 152 young people who completed the questionnaire in this study fell outside the 13–18 year age range, of whom 21 were older and 7 younger, and 31 individuals were aged 17 or 18 years. Table 2 shows the ages of the sample, broken down by clinic. There were 72 males (47% of the sample), [mean age 16.79 ± 2.64; range: 12.8 to 24.0 years] and 80 females (53%), [mean age 15.96 ± 2.17, range: 10.4 to 22.7 years]. Over three-quarters (78%) of the sample were attending school or sixth form college at the time of questionnaire completion, 7% were at university, 12% working, and the remainder unemployed. In order to have sufficient data for the psychometric analyses and to evaluate the questionnaire for the wider age range, it was decided to use the data for all 152 respondents, even those who fell outside the 13–18 age range. Key analyses were re-run on the subset of 13–18 year olds to check that results were similar to those from the full data set, although sample size (124) in this age range was less than optimal.

**Table 2:** The sample of 152 young people who completed the ADDQoL-Teen

**Hospital**	**Male**	**Female**	**Paediatric clinic**	**Adult clinic**	**Mean age**	**Total**
A	16	13	21	8	16.58 ± 1.28	29
B	21	25	38	8	15.80 ± 2.12	46
C	11	13	17	7	18.27 ± 3.50	24
D	8	14	16	6	14.92 ± 1.27	22
E	10	11	11	10	15.11 ± 1.29	21
F	6	4	10	-	19.44 ± 1.32	10

**Total**	72	80	113	39	16.36 ± 2.43	152

### Questionnaire completion rates

Completion rates were very high, providing an indication of the acceptability of the questionnaire to respondents: 'a'/frequency stem items (99.6%); 'b'/bother stem items (99.4%); overview items (98.0%).

### Homogeneity of the patient sample

Initial analyses demonstrated that the six subgroups (recruited from six centres) could be treated as one for the purposes of reliability and factor analyses where a larger N is desirable. Percentage variance accounted for by the loadings on the forced 1-factor analyses was very similar: raw weighted scores (27.52%); standardised z scores for each hospital subgroup recombined (25.62%). Regression analysis found no significant difference between the two sets of loadings: the correlation of 0.987 was close to a perfect 1 (p < 0.001), the constant (0.027) close to zero [t (28) = 1.83, p > 0.05] and the slope (0.916) also close to 1, [t (28) = 32.8, p < 0.0001].

### Descriptive statistics

Frequency analyses of domains indicated that from 1 to 82% of respondents used the *No *– *I do not *(N/A) response option (Table 3). The items with the highest frequency of N/A responses were 17: *miss events *(82%), 14: *sleep away *(80%) and 30: *holidays *(72%). Thus the great majority of respondents did not consider that they missed any events, or sleeping over at a friend's house as a result of their diabetes, nor had their diabetes resulted in going away on British Diabetic Association (B.D.A, now known as Diabetes UK) holidays or weekends or making new friends. The areas of greatest importance/frequency of feeling (Response: *Yes *– *a lot *in the 'a'/frequency stem) were: 5: *insulin *(69%), 9: *finger tests *(49%), 28: *social life *(26%), and 2: *sweets *(25%). The areas where the highest percentage of young people considered they were very much bothered were: 12: *unwell *(25%), 6: *bleed*, 8: *interrupt do *and 19: *high BG *(all 21%), (Table 3). The positive response option (*No *– *it does not bother me*, *I like it*) was used by up to 19% of respondents: 19% liked going to the diabetes clinic, 7% liked finger prick blood tests, 5% liked taking insulin, and 3% liked other people fussing or worrying about them because of their diabetes (items 29, 9, 5, and 1 respectively).

**Table 3:** Descriptive statistics of ADDQoL-Teen domain items

**No:**	**Abbreviation**	**N**	**% N/A**	**% 'a'/frequency: *Yes, a lot*^‡^**	**% 'b'/bother: *very much*^§^**	Weighted impact: Mean ± SD**	**Median [range]**
1	*others fuss*	148	2.6	21.1	9.9	-2.16 ± 2.52	-2 [-9 to 3]
2	*sweets*	112	25.3	24.7	16.0	-3.24 ± 3.24	-2 [-9 to 0]
3	*drink*	80	46.4	6.0	5.3	-2.11 ± 2.31	-1 [-9 to 0]
4	*eat*	117	21.9	16.6	14.7	-2.62 ± 3.09	-1 [-9 to 1]
5	*insulin*	151	0.7	69.1	14.5	-2.21 ± 3.27	0 [-9 to 3]
6	*bleed*	142	6.0	15.9	21.2	-2.39 ± 2.70	-1.5 [-9 to 1]
7	*extra things	87	42.8	7.9	-	1.78 ± 2.80	0 [-2 to 9]
8	*interrupt do*	90	40.0	14.7	20.7	-3.50 ± 3.23	-2 [-9 to 0]
9	*finger tests*	149	0.7	49.0	19.3	-2.16 ± 3.21	-1 [-9 to 3]
10	*control*	87	41.3	16.0	5.4	-1.85 ± 2.90	-1 [-9 to 2]
11	*moody*	125	16.7	19.3	18.4	-2.89 ± 3.04	-2 [-9 to 3]
12	*unwell*	131	13.2	13.8	24.5	-2.93 ± 2.87	-2 [-9 to 3]
13	*out of fix	85	44.1	11.2	-	1.47 ± 2.78	1 [-3 to 9]
14	*sleep away*	30	80.3	2.0	4.6	-2.23 ± 2.75	-1 [-9 to 3]
15	*wake nights*	111	27.0	4.6	18.4	-2.08 ± 2.22	-1 [-9 to 2]
16	*lie in bed*	77	48.3	16.6	14.7	-3.68 ± 3.41	-2 [-9 to 3]
17	*miss events*	27	82.2	2.6	5.9	-2.67 ± 3.10	-1 [-9 to 1]
18	*low BG*	120	20.5	6.6	11.3	-1.71 ± 2.18	-1 [-9 to 3]
19	*high BG*	130	13.2	9.9	20.7	-2.77 ± 2.77	-2 [-9 to 1]
20	*worry future*	93	38.2	17.1	13.9	-3.45 ± 3.28	-2 [-9 to 3]
21	*career*	100	34.9	15.8	20.4	-3.43 ± 3.15	-2 [-9 to 2]
22	*different*	81	46.1	9.9	12.6	-2.72 ± 2.93	-1 [-9 to 0]
23	*not allowed*	105	30.9	10.5	20.4	-3.16 ± 2.99	-2 [-9 to 2]
24	*family life*	89	40.8	9.2	13.2	-2.60 ± 3.11	-1 [-9 to 3]
25	*responsibility*	93	37.7	11.9	6.7	-1.83 ± 2.85	-1 [-9 to 3]
26	*play sport*	96	36.8	11.2	8.6	-2.17 ± 2.86	-1 [-9 to 3]
27	*go toilet*	97	36.2	8.6	11.8	-2.14 ± 2.63	-1 [-9 to 3]
28	*social life*	135	10.6	25.8	17.9	-2.84 ± 3.28	-1 [-9 to 0]
29	*clinic visits*	148	2.0	23.0	2.6	-0.30 ± 2.08	0 [-9 to 3]
30	*holidays	43	71.5	2.6	-	2.40 ± 2.83	2 [-1 to 9]

*positive item.

**max possible range negative items [-9 to 3] and positive items [-3 to 9].

^‡^valid % of *Yes *– *a lot *in response to the 'a'/frequency stem.

^§^valid % of *Yes *– *it/they bother/s me very much *in response to the 'b'/bother stem.

**Figure 2:**
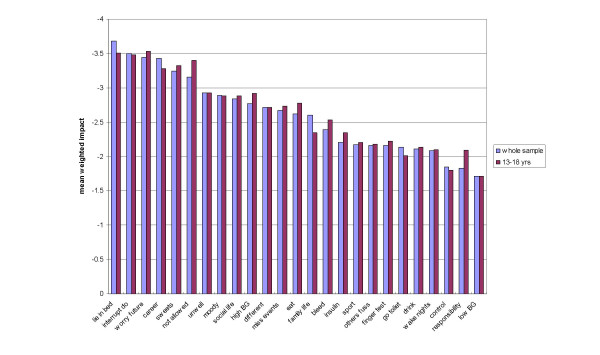
Mean weighted impact scores of the domains of the 25-item ADDQoL-Teen for the whole sample and 13–18 year age group.

As expected, all negative items showed negative weighted impact of diabetes on the domains, whereas positive items indicated positive impact of diabetes on domains. The most severe negative impact of diabetes was felt (in descending order of impact, means in brackets) for 16: *lie in bed *(-3.68), 8: *interrupt do *(-3.5), 20: *worry future *(-3.45), 21: *career *(-3.43) and 2: *sweets *(-3.24) (Fig. 2). The least severe negative impact of diabetes was felt for 29: *clinic visits *(-0.3), 18: *low BG *(-1.71), 25: *responsibility *(-1.83) and 10: *control *(-1.85). Diabetes had the most positive impact on 30: *holidays *(2.4) (noting that this item was only applicable to 28% of respondents) and 7: *extra things *(1.78). Overview items found that although the majority (72.5%) considered that their present QoL was *good *or *brilliant *(mean 1.79), 61.8% felt that having diabetes had a negative impact on QoL (mean -0.83), but 35.6% considered it had no impact on QoL, and 2.6% that the disorder had a positive impact on QoL (Table 4).

**Table 4:** Descriptive statistics of ADDQoL-Teen overview items

**No:**	**Abbreviation**	**N**	**Mean ± SD**	**Median [range]**
A	*present QoL*	149	1.79 ± 0.97*	2 [-2 to 3]
B	*diabetes-dependent QoL*	149	-0.83 ± 0.88**	-1 [-3 to 1]

*max possible range [-2 to 3]; **max possible range [-3 to 1].

### Preliminary factor and reliability analyses of the 30-item ADDQoL-Teen

Preliminary factor and reliability analyses were conducted to determine the number of items in the scale that could be summed into the overall ADDQoL-Teen AWI score. Full results are not provided, but these analyses resulted in the decision not to include five items (items 7, 13, 14, 29, 30) in the summation of an overall scale AWI, for the following reasons. The three positive items (7: *extra things*, 13: *out of fix*, 30: *holidays*) had unsatisfactory loadings, (<0.4), in a forced 1-factor analysis of the 30-item scale. It was decided to omit them from summation of AWI and, as further reliability and factor analyses did not indicate that they formed a subscale, to analyse each of them as separate items. Item 29: *clinic visits *had a relatively low corrected item-total correlation (0.218), reduced the reliability of the whole scale, and had an unsatisfactory forced 1-factor loading (<0.2). Indeed a high percentage reported that they were not bothered by attending clinic (57%) or that they liked it (19%). Item 29 can also be analysed separately. However, item 14: *sleep away *had an unsatisfactory forced 1-factor loading (<0.4), and although it contributed to the overall scale reliability, a very high percentage (80%) regarded the domain as N/A and, of those for whom it was applicable, only 8% found that it impacted *a lot *or *a fair bit *on their QoL. As the domain was covered by 17: *miss events*, it was decided to delete item 14 from the scale.

All further analyses below were conducted on the 25-item scale.

### The 25-item ADDQoL-Teen

#### Internal consistency reliability

Cronbach's alpha was close to the ideal level of 0.9 (0.913, N = 133). All corrected item-total correlations were >0.37, i.e. well above the acceptable minimum. None of the 25 items would increase the alpha coefficient if deleted from the scale (Table 5).

**Table 5:** Reliability analysis of 25-item ADDQoL-Teen (whole sample)

**Item**	**Scale mean if item deleted**	**Scale variance if item deleted**	**Corrected item-total correlation**	**Alpha if item deleted**
1: *others fuss*	-44.67	1412.16	0.4881	0.9102
2: *sweets*	-44.33	1357.63	0.6016	0.9079
3: *drink*	-45.60	1429.77	0.5125	0.9103
4: *eat*	-44.56	1363.22	0.5800	0.9084
5: *insulin*	-44.50	1334.19	0.6447	0.9070
6: *bleed*	-44.37	1391.05	0.5024	0.9099
8: *interrupt do*	-44.56	1364.32	0.5632	0.9088
9: *finger tests*	-44.59	1385.38	0.4451	0.9114
10: *control*	-45.51	1426.39	0.3727	0.9121
11: *moody*	-44.35	1376.36	0.5240	0.9096
12: *unwell*	-44.19	1391.50	0.4842	0.9103
15: *wake nights*	-45.15	1423.99	0.5022	0.9102
16: *lie in bed*	-44.75	1404.51	0.3802	0.9127
17: *miss events*	-46.22	1442.99	0.4281	0.9114
18: *low BG*	-45.28	1430.58	0.4483	0.9109
19: *high BG*	-44.33	1392.77	0.5171	0.9096
20: *worry future*	-44.64	1377.43	0.5054	0.9100
21: *career*	-44.42	1364.17	0.5803	0.9084
22: *different*	-45.20	1362.36	0.7117	0.9062
23: *not allowed*	-44.55	1361.46	0.6364	0.9073
24: *family life*	-45.16	1372.68	0.6134	0.9078
25: *responsibility*	-45.50	1415.25	0.4332	0.9111
26: *play sport*	-45.26	1396.84	0.5138	0.9097
27: *go toilet*	-45.38	1422.21	0.5002	0.9102
28: *social life*	-44.09	1349.43	0.5856	0.9083

Alpha = 0.9129, standardised item alpha = 0.9144 (N = 133).

#### Factor structure

A forced 1-factor Principal Components Analysis indicated that all but one item (16: *lie in bed*) loaded at ≥0.4 (Table 6). However, whilst item 16 loaded slightly low (0.389) it contributed to overall scale reliability and there did not seem sufficient reason to remove it from the scale, especially as descriptive analysis showed this domain to be the most severely impacted by diabetes (mean weighted impact -3.68 ± 3.41). Thus both reliability and factor analyses of the 25-item scale gave support for the calculation of an AWI score by summing the weighted scores of applicable items.

**Table 6:** Forced 1-factor analysis of 25-item ADDQoL-Teen (whole sample)

**Item**	**Loading**
1: *others fuss*	**0.455**
2: *sweets*	**0.631**
3: *drink*	**0.543**
4: *eat*	**0.639**
5: *insulin*	**0.620**
6: *bleed*	**0.581**
8: *interrupt do*	**0.622**
9: *finger tests*	**0.475**
10: *control*	**0.403**
11: *moody*	**0.555**
12: *unwell*	**0.498**
15: *wake nights*	**0.554**
16: *lie in bed*	0.389
17: *miss events*	**0.447**
18: *low BG*	**0.455**
19: *high BG*	**0.534**
20: *worry future*	**0.500**
21: *career*	**0.590**
22: *different*	**0.745**
23: *not allowed*	**0.650**
24: *family life*	**0.646**
25: *responsibility*	**0.415**
26: *play sport*	**0.546**
27: *go toilet*	**0.486**
28: *social life*	**0.630**

% variance	30.4%

#### Subscales

An unforced Principal Components Analysis with Varimax rotation found seven factors (not shown). Items referring to other people/the external world loaded on Factor 1 (e.g. 1: *others fuss*, 21: *career*, 22: *different*, 26: *play sport*, 28: *social life*). Factor 2 was concerned with consumption of food and drink (2: *sweets*, 3: *drink*, 4: *eat*). Items concerning the effects of diabetes and its treatment on the individual loaded on the remaining five factors in a pattern that was difficult to interpret and with some items double loading. The scree plot indicated two factors.

A forced 2-factor analysis gave the clearest factor structure (Table 7). Factor 1 contained items that related to the way diabetes and its treatment affected interactions with others and the "external world". It included 1: *others fuss*, items 2, 3, 4 (consumption of food and drink), and 28: *social life*. Item 27: *go toilet *double loaded, loading slightly higher (0.358) on this factor than on Factor 2 (0.329). Factor 2 contained items connected with diabetes, its treatment and effects on the individual, e.g. 5: *insulin*, 6: *bleed*, 9: *finger tests*, and 11: *moody*. Item 25: *responsibility *double loaded, slightly higher on Factor 2 (0.295) than on Factor 1 (0.293). The factors accounted for 20.7% and 17.0% of the variance respectively.

**Table 7:** Forced 2-factor analyses of the whole sample compared with the 13–18 age group

	**Whole sample**		**13–18 years**	
	
	**Impact-Other**	**Impact-Self**	**Impact-Other**	**Impact-Self**
1: *others fuss*	**.571**		**.545**	
2: *sweets*	**.593**	.277	**.559**	.285
3: *drink*	**.530**		**.518**	
4: *eat*	**.611**	.268	**.645**	.274
5: *insulin*	.354	**.540**	.341	**.542**
6: *bleed*	.285	**.560**		**.575**
8: *interrupt do*	**.511**	.359	**.451**	**.499**
9: *finger tests*		**.480**		**.471**
10: *control*	**.482**		.389	
11: *moody*		**.598**	.302	**.519**
12: *unwell*		**.757**		**.714**
15: *wake nights*	.270	**.538**	.273	**.534**
16: *lie in bed*	**.452**		**.501**	
17: *miss events*	**.516**		**.578**	
18: *low BG*		**.602**		**.664**
19: *high BG*		**.721**		**.697**
20: *worry future*		**.570**		**.527**
21: *career*	**.495**	.329	**.547**	.272
22: *different*	**.664**	.371	**.637**	.382
23: *not allowed*	**.670**		**.654**	
24: *family life*	**.691**		**.713**	
25: *responsibility*	.293	.295		.325
26: *play sport*	**.463**	.298	**.496**	.365
27: *go toilet*	.358	.329	**.412**	
28: *social life*	**.648**		**.610**	

Loadings >0.25 are shown, with all loadings >0.4 in bold typeface.

The best solution seemed to be that the 25-item scale had two subscales, one relating to the effects of diabetes and its treatment on interactions with others and the external world (the "Impact-Other" subscale) and the second to effects on the individual (the "Impact-Self" subscale). Item 25: *responsibility *double loaded slightly higher on Factor 2 than on Factor 1, and it was decided to retain this item in the Impact-Self subscale because taking responsibility for diabetes and its treatment will rest increasingly on the individual child as he/she grows older.

Domains of *others fuss, miss events, career, different, not allowed, family life, play sport *and *social life*, on the Impact-Other subscale, clearly relate to interactions with the others and the external world. The consumption of food and drink very often occurs in a social context. Frequent visits to the toilet (27: *go toilet*) or having to stop an activity to inject insulin (8: *interrupt do*) may cause embarrassment socially as well as being annoying for the individual. The association of the other items on this scale with the external world is also explicable: item 10: *control *refers to the individual taking control of diabetes, with less help from other people; and having to get up early in the morning to test/inject may be a major issue, particularly for teenagers, again making the young person with diabetes feel different from others (16: *lie in bed*).

Eight of the ten items of the Impact-Self subscale clearly relate to the effects of diabetes and its treatment on the individual (domains of *insulin, bleed, finger tests, moody, unwell, wake nights, high BG *and *low BG*. As pointed out above, taking responsibility for treatment may have greater impact on the individual child with increasing age (item 25), at the same time the child with diabetes may worry about his/her own future (item 20).

Internal consistency reliability of the 15-item Impact-Other subscale was very satisfactory (Cronbach's alpha = 0.883, N = 138), but falling short of the optimal alpha of 0.9. All corrected item-total correlations were satisfactory (>0.38) and only one item (16: *lie in bed*) would increase alpha if deleted, and then only by 0.001. Similarly the 10-item Impact-Self subscale also had very satisfactory reliability (alpha = 0.818, N = 142). All corrected item-total correlations were satisfactory (>0.38) and no item would increase alpha if deleted. These analyses confirmed the reliability of the subscales, and gave support for summing the subscale items into their respective subscale total scores.

#### Dealing with missing data

The whole 25-item scale was found to be reliable at alpha ≥ 0.9 with maximum one item of missing data and reliable at alpha ≥ 0.8 with up to 10 items of missing data. We recommend that AWI is calculated as the mean of the completed domains with no more than one item of missing data, if the desired alpha level is 0.9, or up to 10 missing values, if the desired alpha level is set at 0.8, which is very acceptable for most research purposes involving group comparisons. The scale is reliable at >0.7 with up to 15 items missing data but we do not advise calculating AWI with this number of missing items, as questionnaire content may well be distorted. The 15-item Impact-Other subscale was reliable at alpha ≥ 0.8 with maximum four items of missing data, but the 10-item Impact-Self subscale was reliable at alpha ≥ 0.8 with no item of missing data. Higher levels of reliability (alpha ≥ 0.9) are required of measures that are being used to compare an individual's scores across time [19] and for such purposes the full scale score would be needed with no more than one applicable item missing (excluding N/A items).

#### ADDQoL-Teen AWI and subscale scores

Analysis of the data for the whole sample found that mean overall ADDQoL-Teen AWI was -2.39 ± 1.68, mean Impact-Other was -2.44 ± 1.86 and mean Impact-Self was -2.31 ± 1.86, (maximum possible range -9 to 3) implying that young people perceived that diabetes had a negative impact on their QoL, on interactions with others and the external world, and on themselves.

#### Sex differences

There were no significant sex differences in ADDQoL-Teen AWI and subscale scores after a Bonferroni correction requiring significance of p = 0.017 or less for that family of variables. However, the sex difference in Impact-Self approached significance (p = 0.028) on a Mann-Whitney test, with female respondents tending to show greater perceived negative impact of diabetes on self-related factors (-2.6 ± 1.85) than did male respondents (-1.99 ± 1.84). Considering the 25 ADDQoL-Teen items as another group (with Bonferroni correction requiring minimum significance of p = 0.002), sex differences in 6: *bleed *reached significance. Female respondents showed significantly greater perceived negative impact of having bleeding or bruising at site of insulin injection (-3.01 ± 2.92) than did males (-1.65 ± 2.21) [U = 1764.5, p = 0.002, 2-tailed]. Sex differences also approached significance for 20: *worry future *(p = 0.011), and 22: *different *(p = 0.043) and, considering the three positive items as another family of variables, for 7: *extra things *(p = 0.026). Compared with males, females showed a tendency towards greater perceived negative impact of diabetes on feeling different from peers, worries about the future, but greater positive impact on getting extra things because of their diabetes.

#### Correlations with age

Small but significant positive correlations with age were found for AWI and the two subscales (Table 8) indicating lessening impact of diabetes on overall QoL as measured by the ADDQoL-Teen, lessening impact of diabetes on relationships with others and external world (Impact-Other) and on self-related factors (Impact-Self) with increasing age. There were also significant positive correlations with age for the two overview items, indicating improving present QoL with increasing age, and lessening impact of diabetes on QoL. Moderate correlations were found between ADDQoL-Teen AWI and the overview item QB: *diabetes-dependent QoL *(rho = 0.49), and a smaller correlation, as expected, with overview item QA: *present QoL *(rho = 0.34).

**Table 8:** Correlations between ADDQoL-Teen AWI, subscales, overview items and age at completion of questionnaire (whole sample)

	**Age**	**AWI**
ADDQoL-Teen AWI	0.21 (p = 0.01)	
Impact-Other subscale	0.22 (p = 0.006)	0.90 (p < 0.001)
Impact-Self subscale	0.16 (p = 0.043)	0.85 (p < 0.001)
QA: *present QoL*	0.19 (p = 0.02)	0.34 (p < 0.001)
QB: *diabetes-dependent QoL*	0.26 (p = 0.002)	0.49 (p < 0.001)
N (range)	149 – 152	149 – 152

All correlations are 2-tailed non-parametric Spearman's rho, and significant after Bonferroni corrections applied.

### The 13–18 year age group

The mean age of those in the 13–18 year age group was 15.82 ± 1.47, a little less than that of the whole sample (16.36). Mean weighted impact scores of the younger group were very similar to those of the full sample (Fig. 2). The most negatively impacted domains, in descending order (mean ± SD) were: 20: *worry future *(-3.53 ± 3.36), 16: *lie in bed *(-3.51 ± 3.32), 8: *interrupt do *(-3.48 ± 3.33), and 23: *not allowed *(-3.4 ± 3.12). A forced 1-factor analysis of the scores of the 124 teenagers in the 13–18 age range on all 30 items, found support for excluding the same five items from the scale as described above for the whole sample (i.e. the three positive items, and items 14 and 29). All 25 ADDQoL-Teen items loaded >0.4 on a forced 1-factor analysis except 10: *control *and 25: *responsibility *(loading at 0.356 and 0.394 respectively, full results not shown).

Regression analysis found no significant difference between the forced 1-factor loadings for the subset of 13–18 year olds and those for whole sample (N = 152). The correlation of 0.954 was close to 1, the constant (0.027) was close to zero [t (23) = -0.71, p > 0.05] and the slope (1.04) was also close to 1, [t (23) = 15.21, p < 0.001]. This high correlation indicated that data from the whole sample could substitute for that from the narrower age range. Table 7 compares loadings obtained from the forced 2-factor analyses of the 13–18 year age group with those of the whole sample. The loadings are very similar, except that the double loading of 8: *interrupt do *is higher on Impact-Self with the 13–18 year group, perhaps implying that the younger age group may have less responsibility for deciding on whether to interrupt an activity because of their diabetes, and this is seen as impacting more on the self than on others; and 10: *control *loads less than optimally (0.389) on Impact-Other in the 13–18 age group. 27: *go toilet *loads >0.4 on Impact-Other in the 13–18 age group, but double loads with the wider age range.

Cronbach's alpha of the whole 25-item scale was 0.9132, (N = 106) and only 10: *control *would marginally increase alpha if deleted (0.9133). All corrected item-total correlations were satisfactory. The scale was found to be reliable at 0.9 with up to two items missing and reliable at 0.8 with up to 10 items missing. The 15-item Impact-Other subscale had good internal consistency reliability (Cronbach's alpha = 0.887, N = 111) and was reliable at 0.8 with up to four items missing. The 10-item Impact-Self subscale was reliable (alpha = 0.805, N = 114) if no items were missing. All corrected subscale item-total correlations were satisfactory.

Note: Cronbach's alpha for the sample in the 13–18 age range was only marginally lower (by 0.005) than that for the narrow 13–16 age range (0.918, N = 76), again indicating that the addition of respondents aged 17–18 years is not harmful to the questionnaire's reliability.

### Free comments section

The free comments section at the end of the ADDQoL-Teen was used by 49 young people in all. The majority of respondents' comments emphasised a response that they had already made to a questionnaire item. The following areas were mentioned by at least four individuals and are not directly covered in the questionnaire. Consideration will be given to adding further items to cover these new areas in the future:

• The effect of diabetes on patient's lives, and having to organise/plan life around diabetes and its treatment (nine respondents).

• Other people, including healthcare professionals, not understanding diabetes and its effects on the young person's life (five respondents).

• Concerns about weight, and difficulty losing weight (four respondents).

Although 17–18 year olds were not included in the focus groups at the questionnaire design stage, analysis of the free comments showed that only five of the 49 respondents offering comments fell outside the narrower age range of 13–16 years: four young people were aged 17, and one was within a few days of their 13^th ^birthday. However, each of these four 17 year olds commented on a *different *aspect of life with diabetes not already covered by the questionnaire (i.e. there was no salient aspect missing from the questionnaire on which all four commented). If the questionnaire was not suitable for those aged 17–18, and was missing important domains for these older respondents, it is very likely that a greater number of older respondents would have taken the opportunity to comment at this point. We can be reassured therefore that the questionnaire is suitable for the older age group (17–18 years), even though the measure was not specifically piloted with them.

## Discussion

The ADDQoL-Teen is a new child-centred, individualised questionnaire measuring the impact of diabetes and its treatment on the QoL of teenagers. The items not only reflect the concerns of teenagers with this condition, as expressed in interviews and focus groups, but also use teenagers' wording where possible. Twenty-five of the life domains form a scale with excellent internal consistency reliability. Summation of the weighted impact scores from the applicable items into a single score, the ADDQoL-Teen AWI, gives a measure of the Average Weighted Impact of diabetes on the QoL of the individual. There are two subscales: the 15-item Impact-Other subscale, measuring the impact of diabetes and its treatment on interactions with others and the external world, and the 10-item Impact-Self subscale, measuring the impact of diabetes and its treatment on the individual. Both subscales have good internal consistency reliability. The two overview items (QA and QB) provide global measures of an individual's present QoL, and the perceived impact of diabetes and its treatment on their QoL respectively and, as expected, QB has a higher correlation with AWI than QA, as both QB and AWI measure impact of diabetes on QoL. Of the original 30 items, one item, concerning sleeping away from home, was deleted from the scale as it detracted from scale reliability and factor structure, and was not applicable to the great majority of respondents. Four items, three of which concerned potential positive aspects of diabetes such as getting extra things like snacks or treats, either did not load well with the 25 items in the single scale, or detracted from reliability, but can be analysed individually.

Despite the majority describing their present QoL as *good *or *brilliant*, young people perceived overall negative impact of diabetes on QoL (AWI), including negative impact on interactions with others and the external world (Impact-Other), and on themselves (Impact-Self). However, interesting information can also be gleaned by analysing frequencies of individual domains. Domains reported as most severely (and negatively) impacted by diabetes were *lie in bed*, *interrupt do*, *worry future*, *career *and *sweets*. These show the particular concerns of young people about not being able to stay in bed in the morning like many of their contemporaries, owing to the demands of the diabetes treatment regimen, and the way that this treatment regimen interrupts their normal day-to-day activities. Respondents were also looking to the future and were concerned about their career prospects, getting married, having children, and their longer-term health. The impact of diabetes on consumption of carbohydrates was most notable in relation to eating sweets. The usefulness of the questionnaire's bi-polar scale was indicated by the numbers of individuals who chose a positive response: almost a fifth of respondents liked attending their diabetes clinic, and perhaps a surprising number liked taking insulin or doing finger prick blood tests (5% and 7% respectively). It was also interesting to note that concerns about having a low blood glucose level had the least negative impact on QoL of any of the domains, although this aspect of diabetes is of major concern to healthcare professionals.

Some sex differences were found. Girls and young women showed significantly greater perceived impact of experiencing bleeding or bruising at the site of insulin injection, and there was a non-significant tendency for females to show greater perceived negative impact than males with respect to feeling different from peers, and worries about the future, but greater positive impact on getting extra things because of their diabetes. With increasing age, correlations indicated reduced perceived negative impact of diabetes on overall QoL (AWI), on relationships with others and the external world (Impact-Other) and on self-related factors (Impact-Self). Present QoL also improved with increasing age. The moderate correlation between ADDQoL-Teen AWI and the overview item QB: *diabetes-dependent QoL *was too low (rho = 0.49) to allow the single overview item to replace the 25-item scale for most purposes.

Content validity was also good: relatively few respondents mentioned new domains in the free comments section at the end of the questionnaire. However, consideration will be given in the future to adding further items to cover new areas: organising life around diabetes, other people's understanding of the condition, and concerns about excess weight.

The teenagers involved in interviews and focus groups during work to design the questionnaire lived in and around London. However, the respondents in the questionnaire study were from six areas in Britain, and there were clear indications of acceptability to all in terms of very high completion rates, and that neutral, non-regional vocabulary had been chosen. In order to have sufficient data for the psychometric analyses it was necessary to use the data for all 152 respondents, even those who fell outside the age range for which the questionnaire was originally designed (13–16 years). Nevertheless, completion rates indicated the acceptability of the questionnaire to a much wider age range than that for which it was originally intended. This is a valuable outcome, as there is considerable variability in age range at paediatric, adolescent, transition and adult diabetes clinics between different diabetes services in the UK. Indeed the mean age of respondents from one of the centres in the present study, a paediatric clinic, was 19.4 years.

The questionnaire can also be recommended for 13–18 year olds, as analyses performed on the subset of data for this age group found results very similar to those for the full data set. Although the sample size (124) in the 13–18 year age range was less than optimal, the factor structure was clear and very similar to that of the wider age range, and the full 25-item scale and two subscales also had very good internal consistency reliability. There appeared to be some slight differences in mean weighted impact scores between the two groups (Fig. 2). The negative impact of not being allowed to do things because of diabetes was higher in the 13–18 year age group (who had a lower mean age), as was the negative impact on diabetes on eating, and of having to take more responsibility than they would like. As might be expected, those in the 13–18 age group also perceived greater negative impact of not being allowed to do things because of their diabetes, and also for high blood glucose levels (glycaemic control often deteriorates in adolescence [25]). Not being able to lie in bed was the most negatively impacted of all domains for the whole sample, and the second most extreme response for the younger age group. Both groups were concerned about the future and the effects of diabetes on their careers. Moreover, there was no evidence from analysis of free comments that the measure was unsuitable for 17–18 year olds, as only four representatives of this age group took the opportunity to comment here, and no aspect was mentioned by more than one of these older respondents. We would not recommend that the measure is used above the age of 18, unless the cognitive development of the young person seemed to indicate that the equivalent adult measure, the ADDQoL, were unsuitable. However, if a hospital has young people over 18 years in its adolescent clinic, clinicians might welcome a measure that has been found in practice to be suitable for young people above this age cut-off when conducting studies on their patients.

Although physiological measures are used to monitor the treatment of children and young people with diabetes, there are no child-centred, individualised psychological instruments currently in use in paediatric clinics that measure the *impact *of diabetes on children's everyday lives and on their QoL. The ultimate aim of QoL measurement is to improve patients' QoL wherever possible, by taking into account the impact of the treatment regimen and the effects of diabetes on their experience of daily living. Use of the ADDQoL-Teen would facilitate understanding of these issues and would provide healthcare professionals with valuable information about the psychosocial effects of diabetes on teenagers' everyday lives, which will help them consider psychological issues as well as medical outcomes when caring for teenagers with diabetes. Children (and parents) are faced with the day-to-day responsibility for the management of diabetes, and any improvements in QoL, whilst welcome in themselves, may also mean that these young patients will be more likely to follow the planned treatment regimen which will, in turn, help improve control of blood glucose levels and contribute to a reduction of long-term complications of diabetes.

## Conclusions

The internal consistency reliability and some aspects of the validity of the new child-centred, individualised ADDQoL-Teen have been established for young people with diabetes, and the measure may be recommended for use with individual patients. The new questionnaire should help health professionals to consider psychological issues as well as medical outcomes when caring for young people with diabetes. The instrument is also expected to be useful in evaluating new treatments and educational interventions for diabetes in clinical trials.

## Authors' contributions

CB and RJH designed the ADDQoL-Teen, with RJH conducting the interviews and focus groups with teenagers that informed the design of the measure. JD and NJHM conceived and designed the interview study and JD conducted the interviews and collated the questionnaires. CVM carried out the psychometric and statistical analyses of questionnaire data and drafted the manuscript. CB contributed to the interpretation of psychometric analyses, decision-making regarding item selection, and manuscript preparation. All authors read and approved the final manuscript.

## ADDQoL-Teen copyright

For access to and a licence to use the ADDQoL-Teen, contact the copyright holder, Clare Bradley PhD, Professor of Health Psychology, Health Psychology Research, Royal Holloway, University of London, Egham, Surrey, TW20 0EX.

Email: c.bradley@rhul.ac.uk
